# Evaluation of a Sudarshan Kriya Yoga (SKY) based breath intervention for patients with mild-to-moderate depression and anxiety disorders*

**DOI:** 10.1017/S1463423619000045

**Published:** 2019-06-20

**Authors:** Kate Hamilton-West, Tracy Pellatt-Higgins, Farnaaz Sharief

**Affiliations:** 1 Centre for Health Services Studies, University of Kent, Canterbury, Kent, UK; 2 Centre for Health Services Studies, University of Kent, Canterbury, Kent, UK; 3 NHS Medway Clinical Commissioning Group, Manage Your Mind, Chatham, UK

**Keywords:** anxiety, breath, depression, mental health, primary care, yoga

## Abstract

**Aim:**

Research identifies a need for expanded therapeutic options for people with mild-to-moderate depression and anxiety disorders treated within the UK National Health Service (NHS). We aimed to examine potential benefits of a Sudarshan Kriya Yoga (SKY) based breath intervention delivered in this context.

**Background:**

SKY is a structured programme derived from yoga in which participants are taught relaxation and stress-management techniques including body postures, breathing exercises and cognitive-behavioural procedures. Previous research has demonstrated benefits for patients with clinical and non-clinical depression and anxiety. However, SKY has not yet been evaluated as a therapeutic option for patients accessing NHS primary care mental health services.

**Methods:**

We evaluated an existing programme available to NHS patients in South East England. The intervention is community-based and delivered via four weekly ‘stress buster sessions’ (1-h duration), one weekend intensive workshop (2.5 days) and four weekly (90 min) follow-up sessions. Analyses were conducted on existing data [measures of depression (Patient Health Questionnaire-9) and anxiety (generalised anxiety disorder-7)] collected as part of routine care, at the start of the programme and three follow-up assessments.

**Findings:**

Baseline data were available for 991 participants, of which 557 (56.2%) attended at least three weekly workshops, 216 (21.8%) attended the weekend workshop and 169 (17.1%) completed the programme. Statistically significant (*P*<0.05) improvements in depression and anxiety were observed in all three outcome assessments. Clinically meaningful change was observed for 74.6% of participants completing the programme. Findings indicate that SKY has the potential to benefit patient outcomes and could be offered more widely as a therapeutic option. We recommend further research to explore patients’ experiences of the programme, determine the number of sessions necessary for improvement/ recovery, define the population most likely to respond and examine potential cost savings (e.g., reductions in antidepressant prescribing/referrals to secondary care).

Common mental health conditions affect a substantial proportion of the world’s population. A systematic review and meta-analysis including 174 surveys across 63 countries revealed that almost one in five respondents met criteria for common mental disorder during the previous 12 months and 29.2% met lifetime prevalence. Anxiety and mood disorders had the highest 12-month prevalence rates; affecting 1 in 15 and 1 in 20 people annually (Steel *et al*., 2014). Findings from the Global Burden of Disease Study (2010) indicate that depression and anxiety are the leading cause of adult disability worldwide (Whiteford *et al*., 2013). The World Health Organization reports that the burden of mental health disorders is continuing to grow, with significant impacts on health and wellbeing, as well as major social and economic consequences (World Health Organization, 2017).

Consistent with the global picture, statistics for the UK indicate that around one in four adults will experience mental ill health at some point in their lives and at least one in six has a mental health problem at any given time. The total cost of mental health to the economy in England has been estimated at £105 billion, with treatment costs expected to double over the next 20 years. Personal and societal costs are also significant; people with mental health problems are more likely to experience unemployment and homelessness and to live in areas of high social deprivation and they are more likely to have poor physical health, which is due in part to higher rates of risk behaviours, such as smoking, poor diet, physical inactivity, alcohol and substance misuse (Department of Health, 2011).

The Improving Access to Psychological Therapies (IAPT) programme is a national programme developed to improve access to evidence-based treatments for depression and anxiety disorders within the UK National Health Service (NHS). IAPT services are primary care mental health services, which offer psychological therapies recommended by the National Institute for Health and Clinical Excellence (NICE) within a stepped care framework. Following an assessment of psychological needs (step 1), patients with moderate-to-severe depression and some anxiety disorders (e.g., post-traumatic stress disorder) are referred directly to step 3 ‘high intensity’ (HI) interventions (one-to-one psychological therapies), while those with mild-to-moderate anxiety and depression are first offered step 2 ‘low intensity’ (LI) interventions (including guided self-help, exercise and psychoeducation) (Clark, 2011). LI interventions have a number of features which differentiate them from HI treatments including lower complexity, reduced contact time and the use of novel forms of delivery (e.g., telephone sessions, group treatment, computerised cognitive behavioural therapy and provision of self-help material). Patients can be ‘stepped up’ to HI therapies if symptoms do not improve, or there is evidence of increasing need/risk (Bennett-Levy *et al*., 2010; Rodgers *et al*., 2012; Ali *et al*., 2014). For full guidance on assessment and pathways to care, see NICE (2011).

The IAPT programme has been successful in IAPT – more than a million people accessed IAPT services in the first three years, of which 680 000 completed a course of treatment. Of these, around two thirds showed reliable improvement in symptoms and 41% moved to recovery (Department of Health, 2012). Recent data indicate that 39.7% complete treatment, with 65.7% of completers showing reliable improvement and 50.4% moving to recovery (Health and Social Care Information Centre, 2017). However, limitations have also been highlighted. For example, research indicates that only a small proportion of people with depression and anxiety disorders meeting criteria for psychological therapies participate in stepped care programmes when offered. Attrition is a problem and occurs more commonly in the first step, suggesting that failure to respond to LI interventions may discourage further engagement. Researchers have therefore advocated expanded patient choice, including access to different interventions within steps or options regarding intensity of intervention across steps (e.g., timing, frequency and style of treatment sessions) (Kay-Lambkin *et al*., 2010; Seekles *et al*., 2011; Firth *et al*., 2015).

Sudarshan Kriya Yoga (SKY) is a comprehensive programme derived from yoga, which has been delivered by certified instructors to millions of people in 152 countries worldwide. Participants are taught practical relaxation and stress-management techniques including body postures, breathing exercises and cognitive-behavioural procedures. Previous research indicates a range of benefits including improvements in measures of stress, sleep quality, wellbeing, cardiovascular, endocrine and immune function (Janakiramaiah *et al*., 1998; Vedamurthachar *et al*., 2006; Kjellgren *et al*., 2007; Agte *et al*., 2011; Subramanian *et al*., 2012; Toschi-Dias *et al*., 2017). Several studies have demonstrated improvements in clinical and non-clinical depression and anxiety (Janakiramaiah *et al*., 2000; Katzman *et al*., 2012; Seppala *et al*., 2014; Doria *et al*., 2015; Toschi-Dias *et al*., 2017). The potential benefits of offering a SKY based intervention programme as a treatment option within the IAPT stepped-care framework have not yet been evaluated.

## Aim

The aim of the current study was to examine the potential benefits of a SKY based intervention programme for NHS patients meeting criteria for IAPT LI interventions.

## Methods

### Design and procedure

We conducted an evaluation of an intervention programme available to NHS patients with mild-to-moderate depression and anxiety disorders in South East England. Patients access the programme via the following routes:

1. IAPT services refer patients if they are assessed as suitable for LI interventions.
2. GPs refer patients with low-level mental health needs that require support.
3. The programme is free to attend and publicised via a website and information leaflets, so people can also self-refer. There has also been some media coverage and some patients hear of the programme through friends who have attended. They can contact the service directly for enquiries and bookings.

Patients referred to/contacting the service complete a questionnaire before attending in which they provide details of their current health and past medical history. They are accepted onto the programme provided they do not meet the following exclusion criteria: age <16 years, pregnant, suicide risk, psychosis, drug or alcohol addiction, taking lithium/other tranquilising medication used to manage severe mental illness

Data analysis was conducted on existing data collected as part of routine care and stored in accordance with the Data Protection Act. Data for patients entering the programme between 4 January 2013 and 2 July 2017 were included in the analyses. Analyses were conducted on anonymised data and the researchers did not have access to any patient identifiable information.

### Intervention

**Figure 1:**
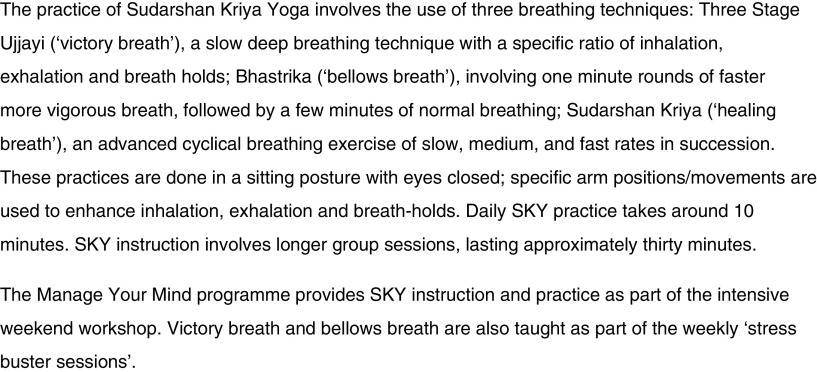
SKY techniques. The practice of Sudarshan Kriya Yoga involves the use of three breathing techniques: three stage Ujjayi (‘victory breath’), a slow deep breathing technique with a specific ratio of inhalation, exhalation and breath holds; Bhastrika (‘bellows breath’), involving 1 min rounds of faster more vigorous breath, followed by a few minutes of normal breathing; Sudarshan Kriya (‘healing breath’), an advanced cyclical breathing exercise of slow, medium and fast rates in succession. These practices are done in a sitting posture with eyes closed; specific arm positions/movements are used to enhance inhalation, exhalation and breath-holds. Daily SKY practice takes around 10 min. SKY instruction involves longer group sessions, lasting ~30 min. The Manage Your Mind programme provides SKY instruction and practice as part of the intensive weekend workshop. Victory breath and bellows breath are also taught as part of the weekly ‘stress buster sessions’.

Manage Your Mind (MYM) is a community programme teaching stress-management and coping skills via four weekly ‘stress buster sessions’ (1 h duration), one weekend intensive workshop (2.5 days) and four weekly (90 min) follow-up sessions. Workshops are facilitated by a certified SKY instructor. Stress buster sessions cover: basic breathing techniques to eliminate stress and tension; guided meditations and processes that calm the mind; knowledge of impacts of lifestyle on health; skills for handling negative emotions and everyday situations and practical advice on improving work and interpersonal relationships. These components are reinforced at the weekend workshop, in which participants are taught SKY techniques (Figure 1), together with cognitive coping and stressor evaluation strategies. Follow-up sessions provide an opportunity for participants to practice techniques with feedback and support from the instructor. Although it is recommended that participants attend the whole programme, this is not a requirement and participants may attend as many sessions as they wish.

### Measures

The nine-item version of the Patient Health Questionnaire (PHQ-9; Kroenke *et al.*, 2001) is used to monitor change in depression symptoms over time, with severity index as follows: 0–4 (no depression), 5–9 (mild), 10–14 (moderate), 15–19 (moderately severe) and 20–27 (severe). Scores of 10 or above are considered to be clinically significant, which is referred to as ‘caseness’. A seven-item measure of generalised anxiety disorder (GAD-7; Spitzer *et al*., 2006) is used to measure severity of anxiety symptoms, with index scores as follows: 0–4 (no anxiety), 5–9 (mild), 10–14 (moderate) and 15–21 (severe). Patients scoring 8 or above are considered to be experiencing clinically significant anxiety symptoms (IAPT, 2011).

### Data analysis

There was evidence of skewness and non-normality in the distribution of both outcome variables GAD-7 and PHQ-9, consequently non-parametric tests were used to analyse the data. GAD-7 and PHQ-9 were analysed using an intention to treat (ITT) analysis and Kruskal–Wallis one way analysis of variance to assess changes from baseline. Last observation carry forward (LOCF) methods were used to account for missing data in the ITT analysis. Sensitivity ITT analysis was conducted using baseline observation carry forward and a per protocol analysis of participants with complete data was also performed. A response bias assessment of demographic data for completers and non-completers was conducted and demographic data summarised.

The number of participants showing reliable improvement/recovery following treatment was calculated following Health and Social Care Information Centre (2014) guidance: reliable improvement requires that any improvement in scores for depression or anxiety between pre and post treatment exceeds the measurement error of the scales (4 or more points for GAD-7 or 6 or more points for PHQ-9) and there is no evidence of reliable deterioration (increase in scores exceeding the measurement error) on either GAD-7 or PHQ-9. An individual is defined as a case if scores are above the clinical threshold for depression and/or anxiety pre-treatment. Movement to recovery requires that scores for an individual with caseness must be below the caseness threshold on both measures at the end of treatment (GAD-7 <8 points and PHQ-9 <10 points). Reliable recovery requires that an individual shows reliable improvement and movement to recovery. To determine whether intervention effects differed according to demographic characteristics, analyses were repeated for males and females separately. Subgroup analyses were also conducted for participants aged 18–35 years, 36–64 years and those aged over 65 years; these age groupings are consistent with those used for reporting IAPT data. Additional subgroup analyses were conducted for participants taking, or not taking medications for depression and anxiety.

### Participants

**Figure 2:**
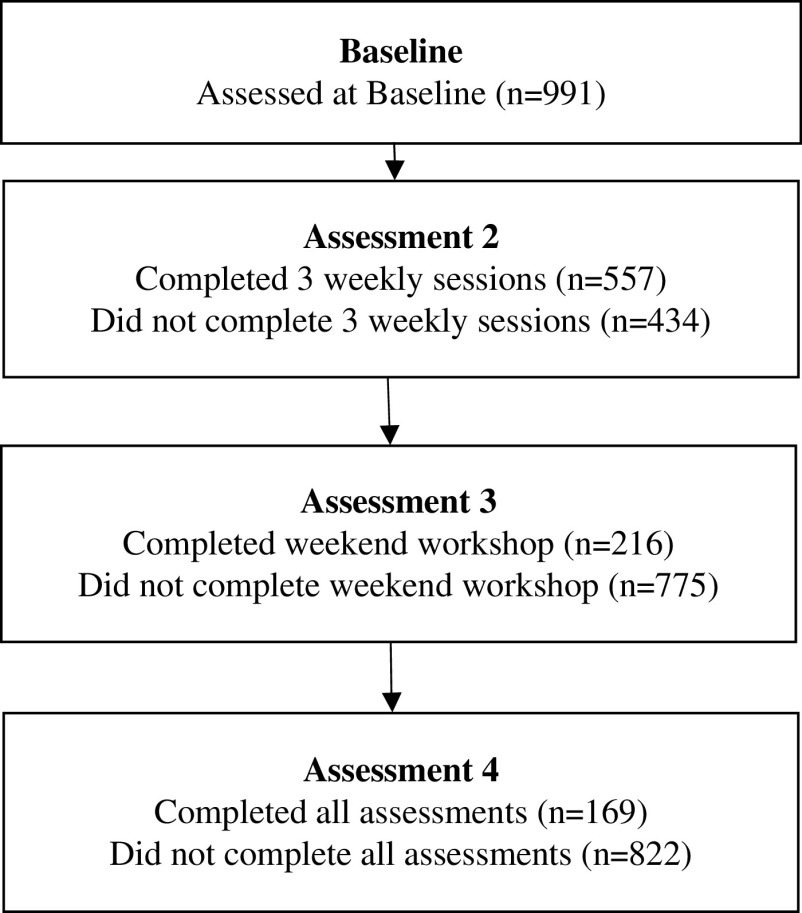
Participant flow diagram

Baseline data were available for 991 participants, of these 254 were male (25.6%) and 737 were female (74.4%). The age of participants ranged from 14 to 80 years and the mean age was 46 years (standard deviation 14.3 years). Pre-treatment mean scores for GAD-7 and PHQ-9 were 10.3 and 10.1, respectively. Scores ranged the full breadth of the scales from 0 to 27 for PHQ-9 and 0 to 21 for GAD-7. At baseline 54% of participants had mild or moderate depression as defined by PHQ-9, and 25% of participants had moderately severe or severe depression. Similarly, 59% of participants had mild or moderate anxiety at baseline and 24% had severe anxiety. Referral data indicated that 689 participants (70.4%) accessed the programme via self-referral, while 179 (18.1%) were referred by a GP and 114 (11.5%) were referred by a counsellor or health professional. There were no obvious differences between these three groups in terms of baseline characteristics. Medication data indicated that 206 participants (20.8%) were taking medications for depression and/or anxiety, while 785 (79.2%) were not. In total 169 participants (17.1%) had complete data at all assessment visits. The number of participants who completed and withdrew from each stage of the programme are shown in the participant flow diagram (Figure 2).

## Results

Summaries of baseline data were compared between completers and non-completers. Table 1 shows summary statistics for age and baseline scores of GAD-7 and PHQ-9; these were similar for completers and non-completers.

**Table 1:** Demographic and baseline data for completers and non-completers

	Statistic	Completers	Non-completers
Females	%	79.3	73.4
Age	Mean	48.3	45.0
	SD	12.0	14.7
	Median	48	45
GAD-7	Mean	10.14	10.33
	SD	5.21	5.39
	Median	10	10
PHQ-9	Mean	9.53	10.27
	SD	5.69	6.35
	Median	9	10

GAD-7=generalised anxiety disorder-7; PHQ-9=Patient Health Questionnaire-9.

**Figure 3:**
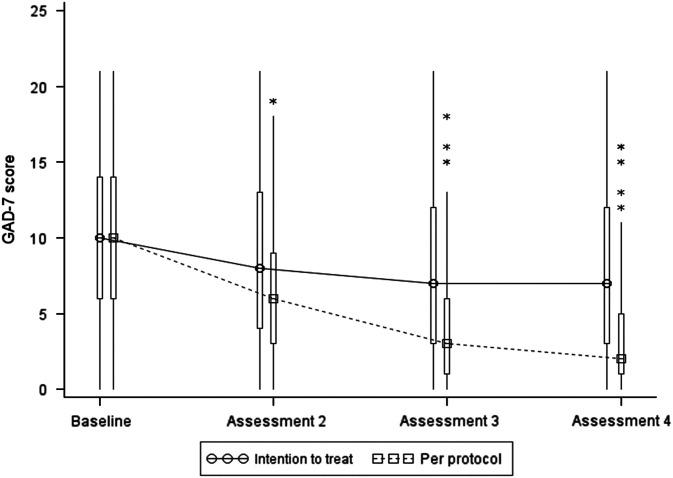
Box and Whisker plot of GAD-7 scores. Note: the boxes represent the inter-quartile range and are intersected at their median point. The whiskers extend to the most extreme point within 1.5 times of the inter-quartile range. The asterisks represent scores which lie outside the whiskers.

Summary statistics based on the ITT LOCF dataset are presented in Table 2 and Figure 3 below. The median and mean values show a decrease from assessment 1 (baseline) in GAD-7 and PHQ-9 at assessments 2, 3 and 4. By the final assessment, only 17% of participants had scores in the moderately severe/severe depression range and only 16% in the severe anxiety range. Almost twice as many participants had scores <5 at the final assessment compared to pre-treatment, indicating they were no longer experiencing depression or anxiety.

**Table 2:** Intention to treat analysis – summary statistics (*n*=991)

Outcome	Statistic	Baseline	Assessment 2	Assessment 3	Assessment 4
GAD-7	Mean	10.30	8.51	7.90	7.81
	SD	5.36	5.45	5.63	5.69
	Median	10	8	7	7
	Interquartile range	8	9	9	9
PHQ-9	Mean	10.14	8.41	7.98	7.83
	SD	6.24	6.20	6.29	6.36
	Median	9	7	7	7
	Interquartile range	10	9	9	10
GAD-7 change from baseline	Mean	0	−1.79	−2.40	−2.49
	SD	0	3.79	4.31	4.32
	Median	0	0	0	0
	Interquartile range	0	3	4	4
PHQ-9 change from baseline	Mean	0	−1.73	−2.16	−2.31
	SD	0	3.86	4.38	4.36
	Median	0	0	0	0
	Interquartile range	0	3	4	4

GAD-7=generalised anxiety disorder-7; PHQ-9=Patient Health Questionnaire-9.

An ITT non-parametric analysis of GAD-7 and PHQ-9 revealed statistically significant differences over time, and pairwise comparisons of baseline data with post-intervention data at assessments 2, 3 and 4 were all statistically significant (*P*<0.001) providing evidence of statistically significant decreases from baseline at all assessments. At the final assessment median anxiety scores were significantly reduced by 30% (*P*<0.001) and median depression scores were significantly reduced by 22% (*P*<0.001). The median change from baseline was zero at all post-intervention assessments for GAD-7 and PHQ-9, indicating that for at least 50% of participants, anxiety and depression scores remained the same or increased following treatment; this suggests the intervention may not have benefit for all participants.

**Figure 4:**
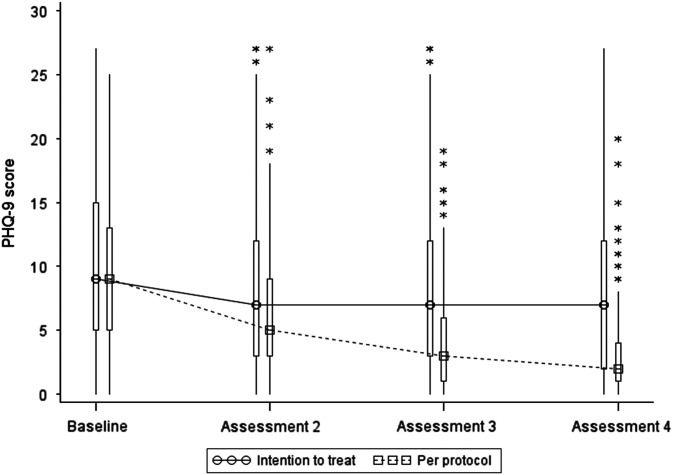
Box and Whisker plot of PHQ-9 scores. Note: the boxes represent the inter-quartile range and are intersected at their median point. The whiskers extend to the most extreme point within 1.5 times of the inter-quartile range. The asterisks represent scores which lie outside the whiskers.

Summary statistics based on the per protocol dataset are presented in Table 3 and Figure 4 below. Inspection of mean and median values indicates a large decrease in both GAD-7 and PHQ-9 at assessments 2, 3 and 4, which is most marked at assessment 4.

**Table 3:** Per protocol analysis – summary statistics (*n*=169)

Outcome	Statistic	Baseline	Assessment 2	Assessment 3	Assessment 4
GAD-7	Mean	10.14	6.46	3.99	3.47
	SD	5.21	4.66	3.89	3.81
	Median	10	6	3	2
	Interquartile range	8	6	5	4
PHQ-9	Mean	9.53	6.14	4.24	3.36
	SD	5.69	4.98	4.17	3.90
	Median	9	5	3	2
	Interquartile range	8	6	5	3
GAD-7 change from baseline	Mean	0	−3.68	−6.14	−6.67
	SD	0	5.17	5.50	5.17
	Median	0	−3	−6	−7
	Interquartile range	0	7	8	7
PHQ-9 change from baseline	Mean	0	−3.38	−5.28	−6.17
	SD	0	5.06	5.96	5.31
	Median	0	−3	−4	−6
	Interquartile range	0	6	7	8

GAD-7=generalised anxiety disorder-7; PHQ-9=Patient Health Questionnaire-9.

A per protocol non-parametric analysis of GAD-7 and PHQ-9 revealed statistically significant differences over time, and pairwise comparisons of baseline data with post-intervention data at assessments 2, 3 and 4 were all statistically significant (*P*<0.001) providing evidence of statistically significant decreases from baseline at all assessments. At the final assessment, median anxiety scores for those who completed the programme were significantly reduced by 80% (*P*<0.001) and median depression scores were significantly reduced by 78% (*P*<0.001).

To assess clinical significance, the IAPT criteria were used to determine the proportion of patients with ‘caseness’ at assessment 1, showing statistically reliable improvement, and the number of participants showing movement to recovery and reliable recovery. The results from this analysis are summarised in Table 4. At baseline 74% of completers had scores above the caseness threshold, this is lower than the most recent IAPT figures (93.5%) indicating that participants in this evaluation have lower severity at baseline than the IAPT referral population. The ITT dataset shows 32.4% of participants with reliable improvement, 23.3% moving to recovery and 22.0% with reliable recovery. Much higher figures were observed for the subset of participants who completed the programme; reliable improvement, movement to recovery and reliable recovery more than doubled in this group compared to the ITT analysis including all participants.

**Table 4:** Clinical significance based on IAPT criteria

Clinical outcome	Per protocol analysis (%)	Intention to treat analysis (%)
Proportion at or above caseness at baseline	74.0	72.1
Proportion showing reliable improvement at assessment 4	74.6	32.4
Proportion moving to recovery at assessment 4	58.6	23.3
Proportion showing reliable recovery at assessment 4	56.2	22.0

IAPT=Improving Access to Psychological Therapies.

All significant differences remained when data were analysed separately for males and females, for those taking, or not taking medications for depression and anxiety and by age group (18–35, 36–64 and over 65 years), indicating that intervention effects do not differ according to participants’ age, gender or concurrent medication. There were higher baseline depression scores for female participants who completed the MYM programme compared to male participants who completed the programme. Those taking medications for depression and anxiety had higher baseline levels of depression and anxiety and showed larger change from baseline to follow-up than those not taking medications. However, the proportionate change was similar across the two groups. The subgroup analysis by age group suggested that participants aged between 18 and 35 years were less likely to complete the programme compared to the other groups; the completion rate for this group was 9.4% compared to 20.6% for those aged 36–64 years and 15.5% for those aged over 65 years.

## Discussion

Improving uptake of treatment programmes for mild-to-moderate depression and anxiety disorders is likely to benefit primary care in a number of ways. For example, teaching patients practical techniques for managing their own wellbeing has the potential to reduce escalation of low-level mental health problems and avoid the need for (more costly) higher intensity interventions, as well as reducing the need for antidepressant medication. Improved mental wellbeing may also benefit physical health and reduce demand on health services. We aimed to evaluate potential benefits of adding a SKY-based breathing intervention to the range of treatments available for patients with mild-to-moderate depression and anxiety disorders.

Findings of this preliminary evaluation are encouraging – the course was attended by males and females, ranging in age from 14 to 80 years; the majority of participants attended at least three weekly sessions and significant improvements in depression and anxiety were observed at the first follow-up. Differences remained statistically significant at the second and third follow-up. Around two thirds of participants completing the programme moved to recovery, with three quarters showing reliable improvement at the final follow-up. Statistically significant differences remained when data were analysed using the last observation carried forward method for handling missing values. Using this method, clinically significant improvement was observed for almost a third of participants. Statistically significant differences also remained when data were analysed separately for males and females, for younger and older adults and for those taking, or not taking medications for depression and anxiety.

Taken together findings suggest that the MYM programme is an effective intervention for improving symptoms of mild-to-moderate depression and anxiety in patients meeting criteria for IAPT LI treatments and that the intervention is suitable for males and females with a wide range of ages. This type of programme may therefore provide a valuable addition to the range of treatment options available to patients with low-level mental health needs.

### Limitations

The study has a number of limitations. First, we do not know why participants chose to access the MYM programme, as opposed to other therapies offered as part of the IAPT programme. It is possible that patients could have accessed other treatments alongside the MYM programme, and/or that participants selected this programme because they had already tried other treatment options; however, we did not have access to this data, so could not consider the effects of previous/concurrent therapies, or explore the reasons underlying uptake of the MYM programme. Without information on previous/concurrent therapies (apart from depression and anxiety medications), it is difficult to compare rates of improvement and recovery with other treatment options. The lack of a control group is a further limiting factor. Hence, it will be important to follow-up this preliminary evaluation with research adopting more rigorous designs, such as randomised controlled trials.

The MYM programme is for people with mild to moderate severity; however, baseline data show that 25% of participants had moderately severe or severe depression and 23% had severe anxiety. In addition, only 72.3% of participants had scores above the caseness threshold at baseline. The distribution of participants in this evaluation is less concentrated in the mild to moderate severity group compared to the IAPT referral population. This also makes it difficult to directly compare outcomes with those reported for the national IAPT programme.

It is also evident that the vast majority of patients entering the programme were female – this is not unusual in studies of SKY and is at least partly attributable to the increased prevalence of depression and anxiety disorders in females (e.g., Doria *et al*., 2015). However, it would be useful to know more about the reasons for increased uptake among female patients. Our data indicate that intervention effects do not differ by gender; hence, if males do access the programme, they are just as likely to benefit as females. Future research could explore acceptability of SKY based interventions in males and identify factors which could be targeted to increase uptake of this type of programme. It would also be useful to further investigate gender differences in baseline depression scores among SKY participants.

A further limitation of the current study is the high volume of missing data resulting from non-completion of the programme. Although the majority of participants attended at least three weekly sessions, a substantial proportion (43.8%) dropped out before the first follow-up and less than a quarter completed the weekend workshop; further attrition was evident at the final follow-up. As noted previously, participants are not required to attend the entire programme and are permitted to attend as many sessions as they wish. However, missing data at follow-up raises challenges for evaluation: the per protocol dataset represents only 17.1% of participants and may be biased as a result, while the ITT LOCF dataset is saturated with estimated values. Both analyses should therefore be interpreted with caution. The completion rate (17.1%) is low compared to recent IAPT data (39.4%); however, it may not be necessary to complete all of the MYM programme to have benefit. Further research is warranted to investigate reasons for non-attendance/withdrawal and to determine whether a minimum number of sessions is necessary for patient benefit. Potential cost savings (e.g., reductions in antidepressant prescribing/referrals to secondary care mental health services) could also be examined.

To advance understanding of the potential benefits of SKY as a treatment option for patients assessed as suitable for IAPT LI interventions, it will also be important to collect qualitative data on participants’ experience of the programme.

## Conclusions

This study is the first to examine potential benefits of a SKY based breath intervention for NHS patients with mild-to-moderate depression and anxiety disorders. Data for patients completing treatment provide evidence of statistically and clinically significant improvement. While these preliminary findings are encouraging, further research is needed to explore patients’ experience of the programme and reasons for uptake/withdrawal. It will also be important to determine the number of sessions necessary for improvement/recovery, define the population most likely to respond and examine potential cost savings.
