# Evaluation of a Sudarshan Kriya Yoga (SKY) based breath intervention for patients with mild-to-moderate depression and anxiety disorders – ADDENDUM

**DOI:** 10.1017/S1463423623000154

**Published:** 2023-05-15

**Authors:** Kate Hamilton-West, Tracy Pellatt-Higgins, Farnaaz Sharief

The author affiliations and funding for this paper were declared at the point of submission and included in the final published version. The journal was made aware of a potential author competing interest in this article after publication. After consultation with the authors, the journal is satisfied that this competing interest does not affect the validity of the research presented in the article. The competing interest disclosure statement is provided below for reader transparency.

*‘The evaluation was funded by a small consultancy grant from Manage Your Mind Community Interest Company (£1685). One of the authors, Dr Farnaaz Sharief, is a director of Manage Your Mind CHC.’*
